# Discovery of Hepatotoxic Equivalent Combinatorial Markers from *Dioscorea bulbifera* tuber by Fingerprint-Toxicity Relationship Modeling

**DOI:** 10.1038/s41598-017-18929-z

**Published:** 2018-01-11

**Authors:** Wei Shi, Cai Zhang, Dongsheng Zhao, Lingli Wang, Ping Li, Huijun Li

**Affiliations:** State Key Laboratory of Natural Medicines, China Pharmaceutical University, Nanjing, China

## Abstract

Due to extremely chemical complexity, identification of potential toxicity-related constituents from an herbal medicine (HM) still remains challenging. Traditional toxicity-guided separation procedure suffers from time- and labor-consumption and neglects the additive effect of multi-components. In this study, we proposed a screening strategy called “hepatotoxic equivalent combinatorial markers (HECMs)” for a hepatotoxic HM, *Dioscorea bulbifera* tuber (DBT). Firstly, the chemical constituents in DBT extract were globally characterized. Secondly, the fingerprints of DBT extracts were established and their *in vivo* hepatotoxicities were tested. Thirdly, three chemometric tools including partial least squares regression (PLSR), back propagation-artificial neural network (BP-ANN) and cluster analysis were applied to model the fingerprint-hepatotoxicity relationship and to screen hepatotoxicity-related markers. Finally, the chemical combination of markers was subjected to hepatotoxic equivalence evaluation. A total of 40 compounds were detected or tentatively characterized. Two diterpenoid lactones, 8-epidiosbulbin E acetate (EEA) and diosbulbin B (DIOB), were discovered as the most hepatotoxicity-related markers. The chemical combination of EEA and DIOB, reflecting the whole hepatotoxicity of original DBT extract with considerable confidential interval, was verified as HECMs for DBT. The present study is expected not only to efficiently discover hepatotoxicity-related markers of HMs, but also to rationally evaluate/predict the hepatotoxicity of HMs.

## Introduction

Herbal medicines (HMs) have been increasingly used among the global population, since they are naturally occurring and beneficial to the health maintenance of human beings^1,2^. Accompanying with wide applications, the potential toxicity of HMs has become a serious issue^3^. It is well-known that, unlike the chemically synthetic drug with high purity, an HM may consist of plenty of complex phytochemicals, which makes the identification of toxic constituents a difficult task^4,5^.

Typically, the traditional approach to identify toxic ingredients from an HM often involves two steps: (1) chemical isolation from the plant extract, and (2) toxic evaluation of each isolates^6,7^. However, the whole procedure is always time consuming and neglects the additive effect of multi-components. As we know, the toxicity of an HM may be caused due to the chemical combination rather than a single constituent^8–11^. Therefore, it is incumbent upon researchers to explore the accurate combinatorial composition of toxic constituents in an HM.

*Dioscorea bulbifera* (DB), belonging to the Dioscoreaceae family, has been largely cultivated in China, Japan, India, Bangladesh and Australia^12–15^. The tuber of DB (DBT), also called “Huang-Yao-Zi” in Chinese, has been clinically employed as a remedy to treat thyroid diseases, leprosy and tumors for several decades^16,17^. Pharmacological studies have shown that DBT possesses anti-tumor, anti-inflammation and goiter inhibitory effects^18,19^. Recently, the associated poisoning cases are occasionally reported in parallel with the rising popularity of those DBT-containing prescriptions in clinical use. Chronic and excessive exposure to DBT has been witnessed to cause liver injury in some patients^20^. Also, the *in vivo* and *in vitro* experimental studies have demonstrated that DBT could induce hepatotoxicity^21–23^. Although the diterpenoid lactones are found to be hepatotoxic^24,25^, the leading role of diterpenoid lactones in evaluating the comprehensive toxicity of DBT remains to be explicitly clarified.

**Figure 1:**
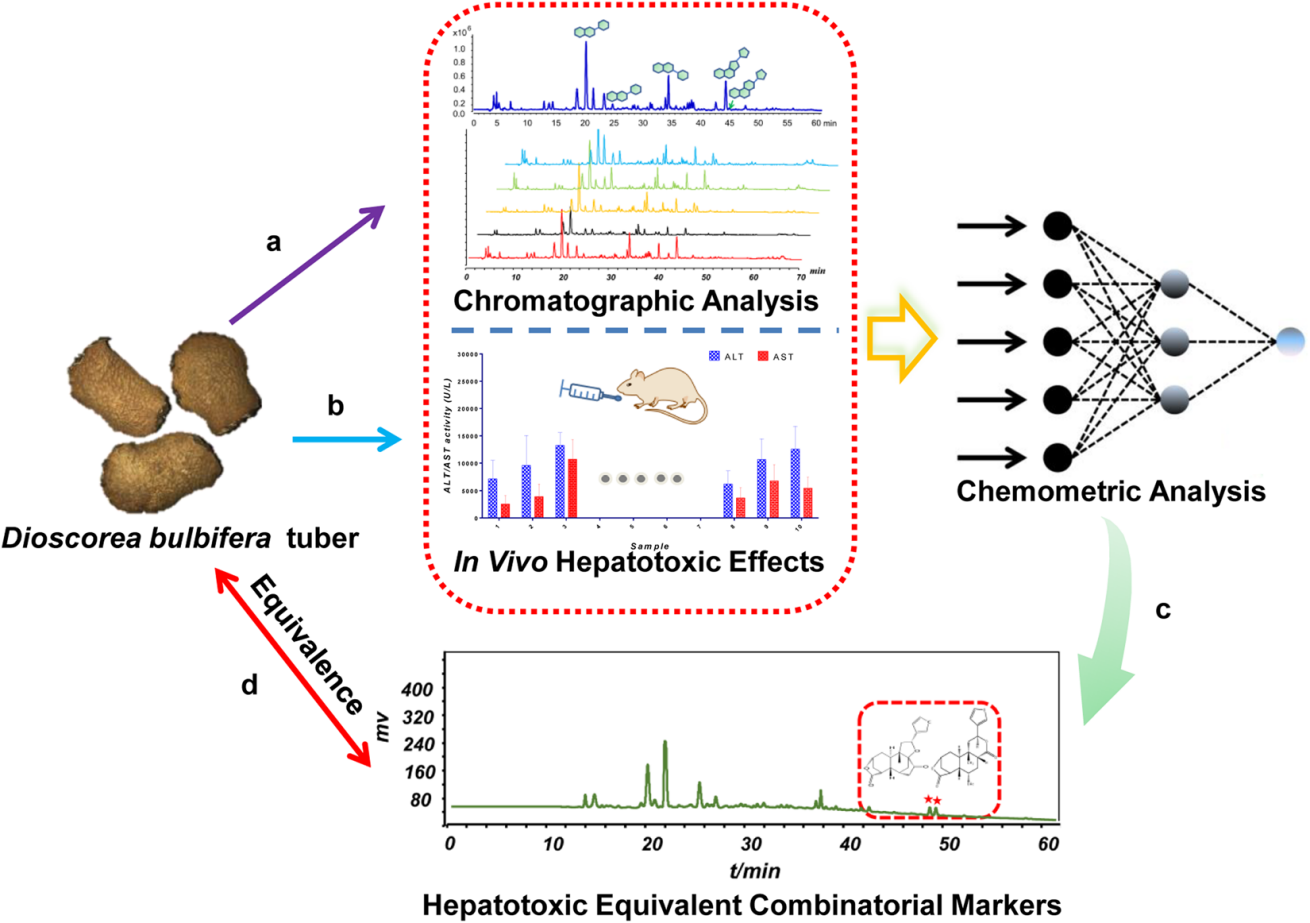
The strategy used to screen the HECMs for the hepatotoxicity evaluation of DBT. (**a**) The chemical profiling and the fingerprint analysis of DBT extracts; (**b**) evaluating the hepatotoxicities of DBT extracts; (**c**) discovering the principal hepatotoxicity markers by three chemometric methods; (**d**) the assessment of hepatotoxic equivalence between candidate HECMs and original DBT extracts. HECMs: hepatotoxic equivalent combinatorial markers; DBT: *Dioscorea bulbifera* tuber.

Very lately, some pioneering works in the field of efficacy evaluation of HM have been launched, where a meaningful term “bioactive equivalent combinatorial components (BECCs)” representative of the holistic effect of an HM^26–29^ has been proposed for the first time. These investigations largely and rationally simplify the screening process of effective components of HMs, avoiding tedious isolation and respective efficacy evaluation. Inspired by the aforementioned studies, we herein attempted to explant the concept of BECCs to discover the “hepatotoxic equivalent combinatorial markers (HECMs)” for the hepatotoxicity evaluation of DBT. This strategy mainly includes the following four steps (Fig. 1): (1) an ultra-high performance liquid chromatography-quadrupole time-of-flight mass spectrometry (UHPLC-QTOF MS) method was established for characterizing the chemical constituents in DBT extract. Meanwhile, an UHPLC-fingerprint analysis with diode array detector was developed for chemical consistency evaluation of 21 batches of DBT samples; (2) the *in vivo* hepatotoxic effects of DBT samples were tested in terms of serum alanine aminotransferase (ALT) and aspartate transaminase (AST) in mice; (3) the fingerprint-toxicity correlation was modeled by multivariate statistical analysis including partial least squares regression (PLSR), back propagation-artificial neural network (BP-ANN) and cluster analysis, the hepatotoxicity-related constituents responsible for the whole toxicity of DBT were screened out; (4) the chemical combination of two diterpenoid lactones was discovered and confirmed as HECMs of DBT, which could be applied to estimate the potential toxicity of DBT samples.

## Results

### Chemical profiling of DBT extract

**Figure 2:**
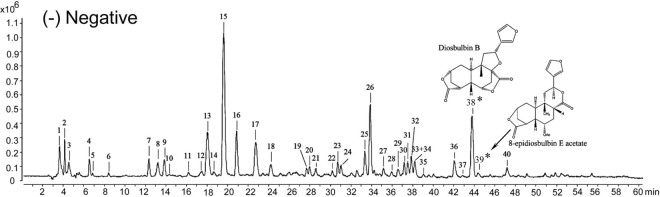
The typical total ion chromatogram of DBT extract by UHPLC-QTOF MS analysis in negative ion mode (**S01**). The peak numbers were in accordance with the compound numbers in Table 1. *HEMCs.

In order to characterize the chemical constituents in DBT extract, an UHPLC-QTOF MS method was established. Both positive and negative ion modes of mass spectra were optimized, and the obtained data suggested that the signal in the negative mode provided higher sensitivity and satisfactory accuracy with all mass errors less than 5 ppm (Fig. 2 and Fig. S1). Based on the retention time (RT), MS and MS/MS spectra data in negative ion mode, a total of 40 compounds, including 20 flavonoids, 7 diterpenoid lactones, 5 organic acids, 2 phenanthrenes and 1 sugar in DBT extract (**S01**) were characterized by the comparison with available reference compounds and previous reports^30–32^. Typical total ion chromatogram of DBT extracts in negative ion mode is shown in Fig. 2. The detailed information such as RT, formula and fragment ions are illustrated in Table 1.

**Table 1:** Characterization of constituents in DBT extract (**S01**) by UHPLC-QTOF MS analysis in negative ion mode.

Peak No.	RT (min)	Proposed molecular formula	Experimental *m/z*	Calculated *m/z*	Error (ppm)	Identification	(−) ESI-MS/MS *m/z*
1	3.740	C_12_H_22_O_11_	341.1092 [M-H]^−^	341.1089	−0.77	Sucrose	59.0118, 89.0221, 101.0238
2	4.329	C_7_H_10_O_5_	173.0449 [M-H]^−^	173.0455	3.72	Shikimic acid	93.0355, 43.0190, 65.0386, 55.0190
3	4.623	C_4_H_6_O_5_	133.0139 [M-H]^−^	133.0142	2.59	Malic acid	43.0179, 71.0111
4	6.683	C_4_H_6_O_4_	117.0190 [M-H]^−^	117.0193	2.81	Succinic acid	99.0015, 73.0295
5	6.983	C_6_H_10_O_5_	161.0451 [M-H]^−^	161.0455	2.76	—	99.0417, 57.0351
6	8.512	C_7_H_6_O_5_	169.0138 [M-H]^−^	169.0142	2.63	Gallic acid	125.0238, 79.0169, 51.0253
7	12.384	C_15_H_14_O_7_	305.0665 [M-H]^−^	305.0667	0.58	Epigallocatechin	179.0453, 165.0099, 137.0229, 125.0238
8	13.199	C_30_H_26_O_13_	593.1287 [M-H]^−^	593.1301	2.30	Kaempferol-3-*O*-β-*D*-galactoside	467.0886, 407.0725, 289.0659, 177.0178, 125.0215
9	13.913	C_7_H_6_O_4_	153.0188 [M-H]^−^	153.0193	3.46	Protocatechuic acid	109.0286
10	14.320	C_30_H_26_O_13_	593.1290 [M-H]^−^	593.1301	1.79	Kaempferol-3-*O*-β-*D*-glucoside	423.0497, 201.0583, 179.0315, 125.0226
11	16.358	C_8_H_14_O_6_	205.0717 [M-H]^−^	205.0718	0.30	—	143.0747, 115.0731
12	17.471	C_30_H_26_O_12_	577.1347 [M-H]^−^	577.1351	0.78	Catechin dimers	407.0769, 289.0701, 245.0791, 161.0207, 125.0229
13	18.091	C_30_H_26_O_12_	577.1342 [M-H]^−^	577.1351	1.64	Catechin dimers	407.0769, 289.0701, 245.0791, 161.0207, 125.0229
14	18.702	C_45_H_38_0_18_	865.1961 [M-H]^−^	865.1985	2.81	Catechin trimers	407.0807, 289.0719, 125.0198
15	19.721	C_15_H_14_O_6_	289.0724 [M-H]^−^	289.0718	−2.20	Catechin	151.0368, 137.0241, 123.0429, 109.0289, 97.0289, 57.0348
16	20.944	C_11_H_12_O_6_	239.0560 [M-H]^−^	239.0561	0.47	—	177.0522, 149.0541,107.0485
17	22.778	C_30_H_26_O_11_	561.1392 [M-H]^−^	561.1402	1.84	Catechin dimers	407.0718, 289.0714, 245.0802, 125.0252
18	24.204	C_15_H_14_O_6_	289.0720 [M-H]^−^	289.0718	−0.82	Epicatechin	159.0424, 137.0229, 123.0448, 109.0298, 57.0344
19	27.669	C_21_H_20_O_13_	479.0830 [M-H]^−^	479.0831	0.24	Myricetin 3-*O*-galactoside	316.0189, 271.0220
20	27.975	C_21_H_20_O_13_	479.0826 [M-H]^−^	479.0831	1.07	Myricetin 3-*O*-gluctoside	316.0162
21	28.586	C_30_H_26_O_11_	561.1389 [M-H]^−^	561.1402	2.38	Catechin dimers	289.0685, 245.0884, 125.0212
22	30.239	C_16_H_16_O_7_	319.0829 [M-H]^−^	319.0823	−1.79	—	225.1468, 194.1215,119.0630
23	30.726	C_21_H_20_O_12_	463.0880 [M-H]^−^	463.0882	0.43	Hyperoside	316.0202, 271.0249,214.0204
24	31.134	C_21_H_20_O_12_	463.0876 [M-H]^−^	463.0882	1.29	Isoquercitrin	316.0226, 271.0229, 242.0167
25	33.477	C_14_H_12_O_4_	243.0662 [M-H]^−^	243.0663	0.34	9,10-Dihydro-2,3,5,7-phenanthrenetetraol	226.0637, 213.0554, 197.0576, 173.0587, 159.0439
26	33.885	C_17_H_14_O_7_	329.0667 [M-H]^−^	329.0667	−0.07	Caryatin	271.0227, 199.0380, 107.0118
27	35.210	C_14_H_10_O_4_	241.0506 [M-H]^−^	241.0506	0.13	2,4,5,6-Phenanthrenetetrol	213.0500, 196.0474, 167.0397, 151.0491
28	35.923	C_19_H_20_O_7_	359.1133 [M-H]^−^	359.1136	0.91	Diosbulbin M	179.0905, 139.0762, 109.0277
29	36.636	C_15_H_10_O_8_	317.0301 [M-H]^−^	317.0303	0.60	Myricetin	179.0028, 151.0025, 137.0234, 107.0134
30	37.248	C_19_H_22_O_7_	361.1286 [M-H]^−^	361.1293	1.87	Diosbulbin L	327.1255, 299.1317, 261.1101, 152.0473, 109.0270
31	37.553	C_17_H_14_O_6_	313.0713 [M-H]^−^	313.0718	1.47	3,5-Dimethoxykaempferol	298.0446, 270.0563, 255.0236, 241.0492, 151.0057
32	37.961	C_19_H_22_O_7_	361.1286 [M-H]^−^	361.1293	1.87	Diosbulbin C	315.1295, 262.1131, 175.1128,123.0821
33	38.267	C_18_H_16_O_7_	343.0808 [M-H]^−^	343.0823	4.44	3,5,3'-Trimethoxyquercein	285.0336, 270.0123, 257.0403
34	38.267	C_28_H_20_O_8_	483.1069 [M-H]^−^	483.1085	3.39	—	255.0717, 241.0513, 211.0389
35	39.039	C_19_H_22_O_6_	391.1412 [M + COOH]^−^	391.1398	2.64	Diosbulbin G	368.1226, 270.0113, 240.0419
36	42.139	C_15_H_10_O_7_	301.0349 [M-H]^−^	301.0354	1.58	Quercetin	273.0421, 151.0020, 121.0272
37	42.954	C_19_H_20_O_6_	343.1179 [M-H]^−^	343.1187	2.36	Diosbulbin D	299.1292, 271.1340, 255.1304, 161.0961
38(*)	43.747	C_19_H_20_O_6_	389.1255 [M + COOH]^−^	389.1242	−3.80	Diosbulbin B	343.1139, 316.1432, 299.1255, 259.1026, 122.1503
39(*)	44.493	C_21_H_24_O_7_	423.1234 [M + Cl]^−^	423.1216	−4.63	8-Epidiosbulbin E acetate	377.2653, 232.0370, 174.0621
40	47.234	C_15_H_10_O_6_	285.0407 [M-H]^−^	285.0405	−0.83	Kaempferol	239.0308, 155.0483, 93.0326

*HEMCs.

### Fingerprint analysis of DBT extracts

**Figure 3:**
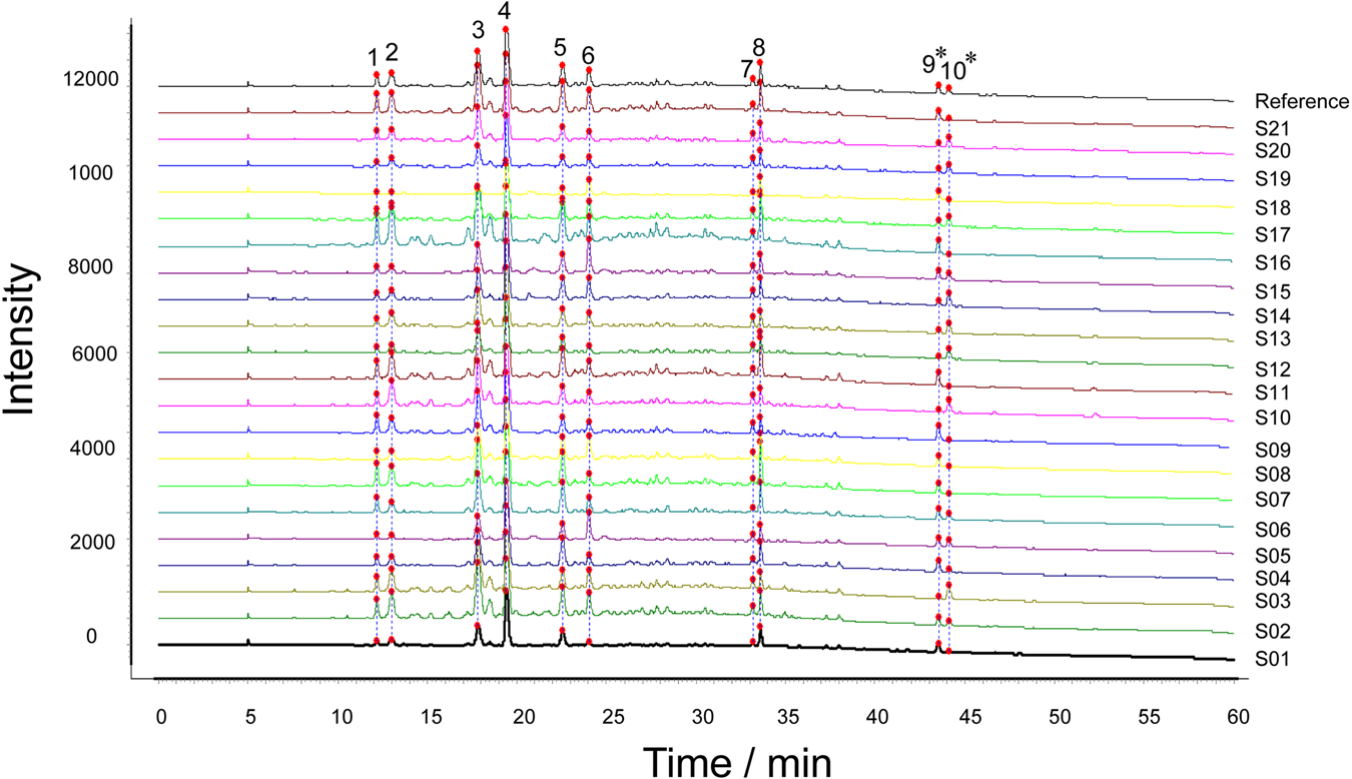
UHPLC fingerprints and common peaks of 21 batches of DBT extracts (**S01**–**S21**). The peaks were identified as epigallocatechin (**1**), kaempferol-3-*O*-β-*D*-galactoside (**2**), catechin dimers (**3**), catechin (**4**), catechin dimers (**5**), epicatechin (**6**), 9,10-dihydro-2,3,5,7-phenanthrenetetraol (**7**), caryatin (**8**), DIOB (**9**), EEA (**10**). *HEMCs.

**Figure 4:**
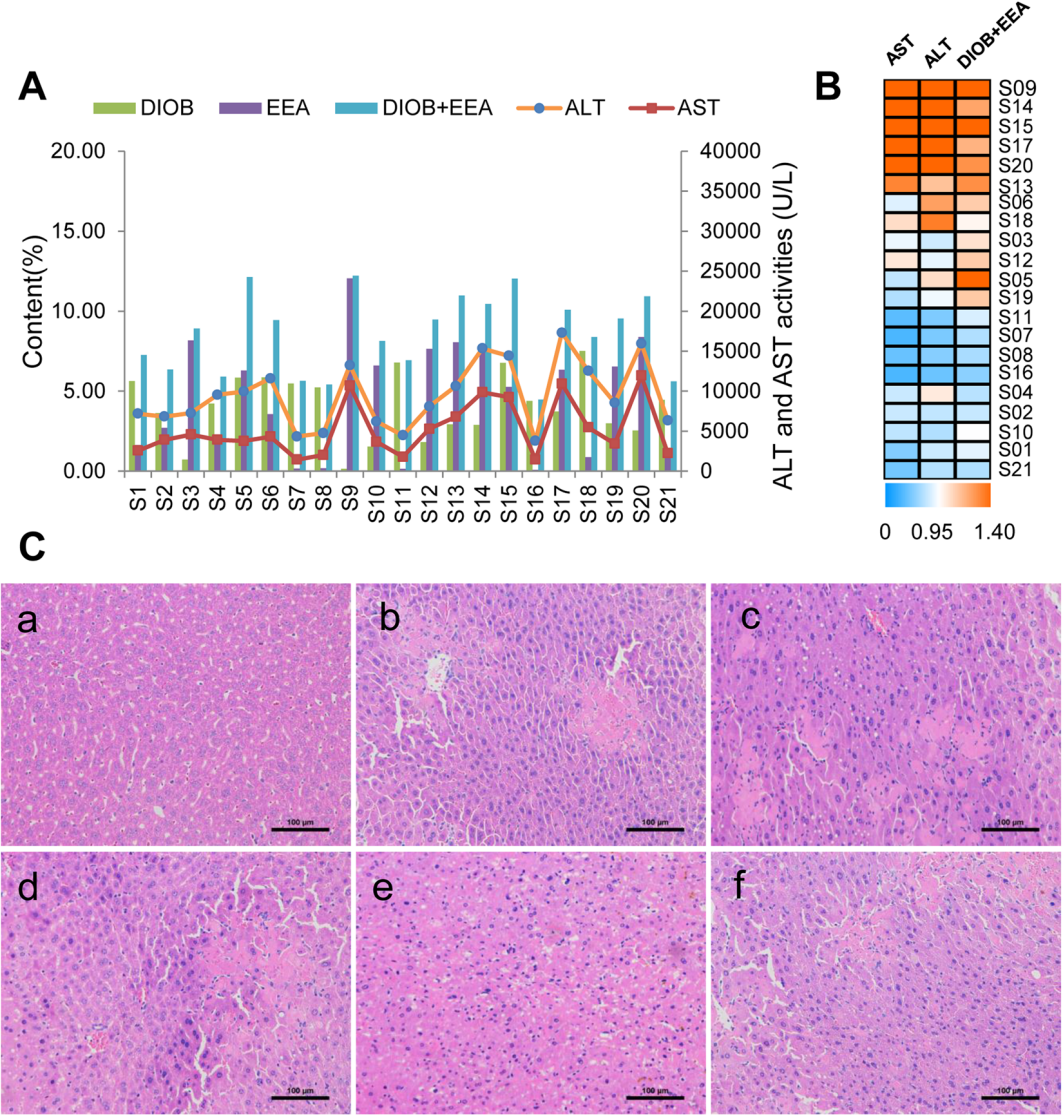
(**A**) Content of EEA and DIOB and the serum ALT and AST activities of 21 batches of DBT extracts (**S01**–**S21**, 2 g/kg). (**B**) The heat map of the combination of DIOB and EEA contents and the serum ALT/AST levels, the shade of colors meant different concentration levels of the combination of these two compounds and ALT/AST. The orange or bluer the color were the higher or lower the relative concentration level. (**C**) Histological observation of DBT extracts induced liver injury. Control (a); b, c, d, e and f were representative images show H&E staining of liver tissue from experimental groups. (Original magnification: ×200).

In order to validate the developed method, the analytical performance test was evaluated. The results showed the relative standard deviations (RSDs) of precision, stability and repeatability were no more than 4%, indicating the analysis was reliable and repeatable. The fingerprints of DBT extracts were established by UHPLC under the optimal conditions (See Fig. 3). Ten common peaks were defined from the fingerprint, which accounted for above 80% of the overall peak areas. Comparing with the chemical profiling data, the peaks were identified as epigallocatechin (**1**), kaempferol-3-*O*-β-*D*-galactoside (**2**), catechin dimers (**3**), catechin (**4**), catechin dimers (**5**), epicatechin (**6**), 9,10-dihydro-2,3,5,7-phenanthrenetetraol (**7**), caryatin (**8**), diosbulbin B (DIOB) (**9**), 8-epidiosbulbin E acetate (EEA) (**10**). The contents of peaks **9** and **10** in DBT samples were quantified (data are shown in Fig. 4(A)).

The similarity results of 21 batches of DBT samples are presented in Table S1, and the similarity values fell within the range of 0.919–0.996. The results of common peak areas are listed in Table S2. As is evidenced from chemical profile data and similarity values among DBT extracts, it is difficult to discriminate the randomly selected samples only by fingerprint analysis.

### Hepatotoxicity of DBT extracts

The levels of serum ALT and AST activities are used as biomarkers for the early acute liver injury, and obvious elevation of these enzymes reflects the damage of hepatic function^33,34^. To assess hepatotoxicity of DBT extract, serum ALT and AST in mice treated with DBT extracts were detected. It was noted that a dose of 2 g/kg DBT extracts resulted in serum ALT/AST levels significantly increased (*p *< 0.01), comparing with 32 ± 6 U/L (ALT) and 83 ± 15 U/L (AST) in mice treated with vehicle (Fig. 4(A,B)). In addition, severe liver damage appeared as hepatic cell necrosis, inflammatory cell infiltration and local spotty necrosis by histopathologic analysis (Fig. 4(C)). These findings indicated the mice administered DBT extracts at 2 g/kg caused potential hepatotoxicity.

In comparison with the results from fingerprint analysis of DBT extracts, the hepatotoxicities of these samples showed different ALT and AST values as 3775–17308 U/L and 1434–11938 U/L, respectively (Fig. 4A). Among all 21 bathes of DBT extracts, samples **S06**, **09**, **13**, **14**, **15**, **17**, **18** and **20** exerted the strongest hepatotoxic effects with the serum ALT activities over 10636 ± 3830 U/L and AST activities over 4310 ± 2495 U/L, followed by samples **S01**, **02**, **03**, **04**, **05**, **10**, **12**, **19** and **21** with the serum ALT (6215 ± 2375 to 9961 ± 3947 U/L) and AST (2237 ± 1582 to 5283 ± 3392 U/L). As expected, samples **S07**, **08**, **11** and **16** showed weak hepatotoxicities.

### Discovery of principal hepatotoxic markers by PLSR, BP-ANN and cluster analysis

In the present study, we tried to establish the fingerprint-hepatotoxicity relationship model, and three chemometric methods, including PLSR, BP-ANN and cluster analysis were applied to screen the possible hepatotoxic markers from DBT extracts.

PLSR is a particular type of multivariate analysis using the two-block predictive PLS model to find the relationship between two matrices, *X* and *Y*^35^. Here, a PLSR model to correlate hepatotoxicity and chromatographic data was constructed with the 21 batches of DBT extracts. The parameter *R*-squared and adjusted *R*-squared of the model were 0.902 and 0.576 respectively, which indicated that PLSR was appropriate in modeling fingerprint-hepatotoxicity correlation. The importance of the *X*-variables for a model could be evaluated by variable importance for the projection (VIP) values (usually with a threshold >1.0). The VIP values of ten peaks are given in Table 2. As observed, the VIP values of peaks **9** and **10** were higher than 1.0 (1.025 and 1.798, respectively), indicating that DIOB and EEA might induce liver injury.

**Table 2:** The MIV between common peaks and VIP values by BP-ANN and PLSR models.

Analyte	Peak 1	Peak 2	Peak 3	Peak 4	Peak 5	Peak 6	Peak 7	Peak 8	Peak 9(*)	Peak 10(*)
VIP value	0.781	0.966	0.861	0.767	0.830	0.783	0.900	0.851	1.025	1.798
BP-ANN MIV	−0.256	−0.254	0.185	0.150	0.200	−0.070	−0.190	0.001	0.255	0.342

*HEMCs.

**Figure 5:**
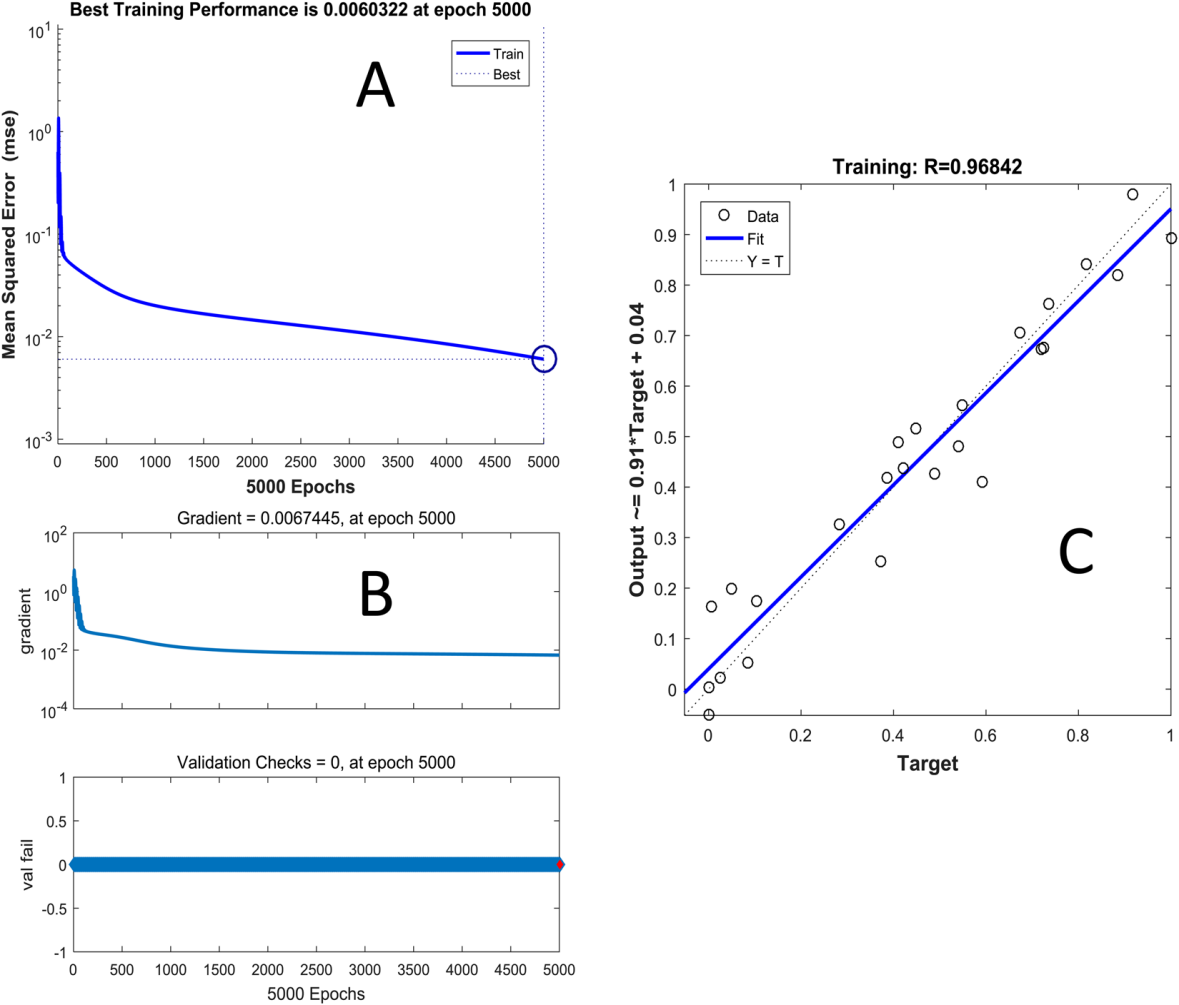
(**A**) The plot of error mean squared in training the BP-ANN, (**B**) the model performance function, through the magnitude of the gradient (upper), and the number of validation checks (down), (**C**) and the regression plot to validate the network trained.

Furthermore, a nonlinear BP-ANN mathematical model was applied to clarify the fingerprint-hepatotoxicity relationship. The model contained three layers and three knots, and the iteration times were 5000. All data sets were from the 21 batches of DBT extracts samples. Mean impact value (MIV) is one of the best indexes reflecting changes of the weights matrix and evaluating the correlation of variables in the neural network^36,37^. In general, the MIV can be described as:

1 $$MIV=\frac{IV}{n}$$

where *n* represents the number of observations. After training on set data, MIVs of the ten common peaks are list in Table 2. The mean squared error (MSE) of training, the magnitude of the gradient and the correlation coefficient (*R*) are depicted in Fig. 5. In line with PLSR modeling results, compounds **9** and **10** had highest coefficient values (0.255 and 0.342, respectively) among all common peaks.

**Figure 6:**
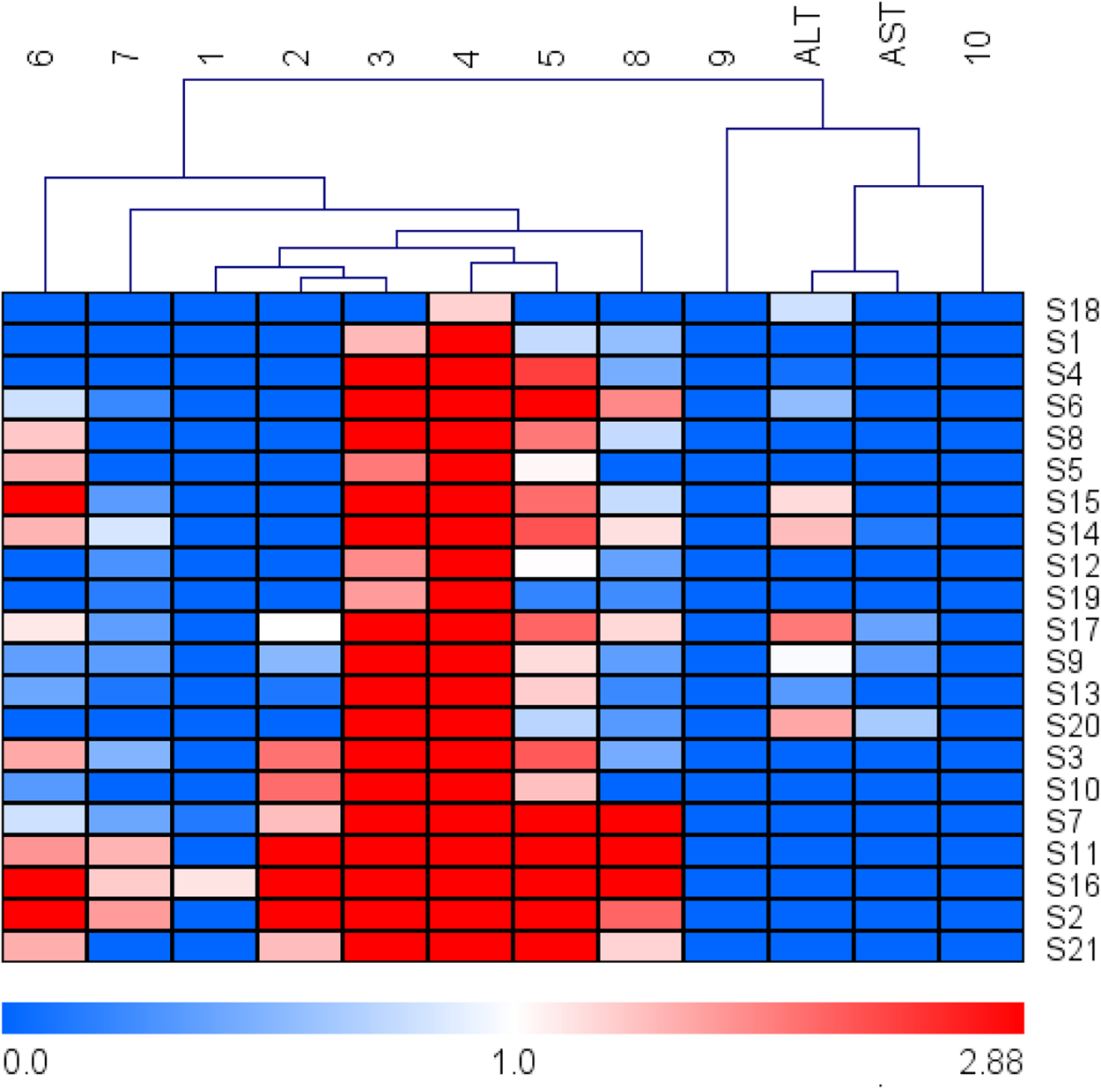
The heat map of ten common peaks and the serum ALT/AST levels, the shade of colors meant different concentration levels of a chemical constituent and ALT/AST. The orange or bluer the color were the higher or lower the relative concentration level.

Finally, heat map was performed to obtain more penetrating understanding of this relationship. The relative concentration trend between the common peaks and serum ALT/AST levels in all test samples is illustrated in Fig. 6. The cluster analyses also suggested that peaks **9** and **10** were more responsible for the hepatotoxicity.

Summing up the results from the extended PLSR, BP-ANN and cluster analysis, DIOB and EEA were finally discovered as the main hepatotoxic compounds in DBT extract. Accordingly, these two compounds were tentatively assigned as candidate HECMs accounting for the whole hepatotoxicity of original DBT extracts.

### Assessment of hepatotoxic equivalence between candidate HECMs and original DBT extracts

**Figure 7:**
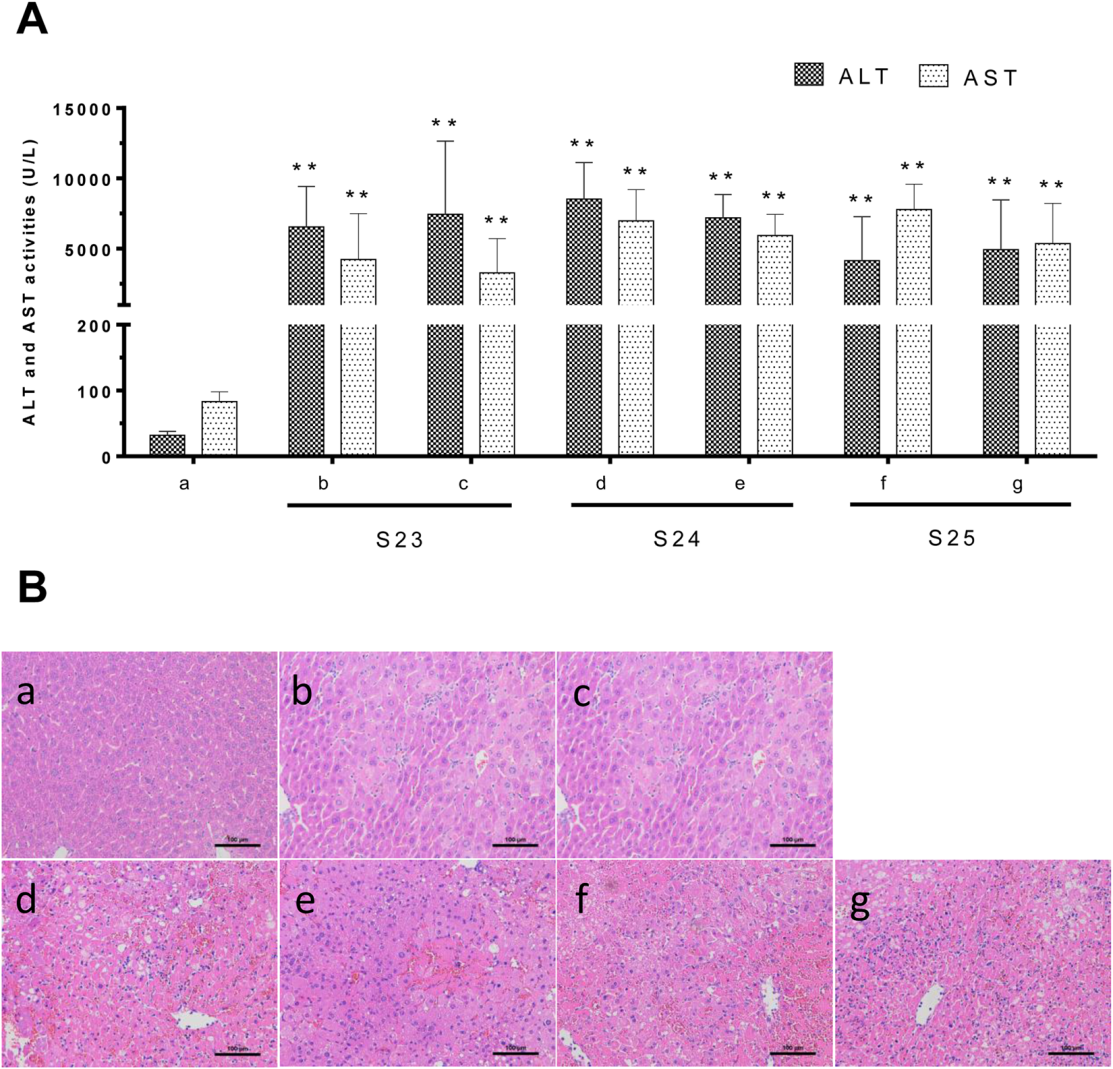
(**A**) Change of ALT/AST activities in mice serum. Mice were treated with vehicle (a), DIOB + EEA-**S22** (b, 660 + 706 mg/kg), **S22** (c, 2 g/kg), DIOB + EEA-**S23** (d, 457 + 645 mg/kg), **S23** (e, 2 g/kg), DIOB + EEA-**S24** (f, 1260 + 116 mg/kg), **S24** (g, 2 g/kg). The mice were sacrificed 36 h after the administration, and the serum ALT and AST levels were measured. **p < 0.01 were considered significantly different. (**B**) Histological observation of samples induced liver injury. Original magnification was 200× for each sample.

The contents of DIOB and EEA in DBT extracts of **S22**–**S24** are summarized in Table 3, and the hepatotoxicities of original DBT extracts and candidate HECMs are shown in Fig. 7.

**Table 3:** DIOB and EEA of calibration curves, LOD, LOQ and Markers contents (%) in three batches of DBT.

Analyte	Linear regression data			LOD (μg/mL)	LOQ (μg/mL)	Contents (%)		
	Regression equation	Test range (μg/mL)	R^2^			S22	S23	S24
DIOB	Y = 10185812.57X + 367287.09	78.77–2520.60	0.9993	5.08	13.10	6.60	4.57	12.60
EEA	Y = 10924180.01X–4967.65	20.55–657.60	0.9998	3.91	15.39	7.06	6.45	1.16

In the hepatotoxic equivalence assessment, both original DBT extracts and candidate HECMs caused significant elevations of serum ALT and AST (*p *< 0.01) (Fig. 7(A)); histopathologic analysis (*n* = 5 per group) revealed local spotty necrosis and inflammatory cell infiltration in the liver of mice given original DBT extracts and candidate HECMs (Fig. 7(B)). The 90% confidence interval (CI) for the hepatotoxicity of candidate HECMs in ALT and AST tests were 99.9–125.6%, 83.4–143.3%, 83.7–121.4% and 93.0–136.9%, 87.4–135.6%, 101.5–117.7%, respectively.

## Discussion

Chemical profiling of HMs is always the prerequisite task for discovery of bioactive compounds^38,39^. Flavonoids and diterpenoid lactones are reported as two main types of ingredients in DBT. Flavonoids are phenolic substances abundantly presented in the plant kingdom, numerous studies have demonstrated their health-promoting properties^40^. In contrast, diterpenoid lactones in DBT are found to be hepatotoxic. Actually, the parent diterpenoid lactones do not appear to be hepatotoxic, while the metabolic activation of the furan ring by cytochromes P450 (CYP450) is the key procedure of acute liver injury^25,41–43^. Considering low CYP expression in cell lines^44^, the *in vivo* animal experiments were therefore conducted to evaluate the potential hepatotoxicity of the DBT extracts.

Similarity analysis of chromatographic fingerprint is well-recognized as a useful measure to evaluate the batch-to-batch chemical consistency of HMs^45–48^. The chemical similarities among 21 batches of DBT extracts were measured by the fingerprint analysis. It was noted that all the similarity values were higher than 0.90, which suggested very little chemical fluctuation existed among those samples. Conversely, the hepatotoxic effects of DBT extracts on mice highly varied. The inconsistence between chemical composition and hepatotoxicity indicated that it is urgent to establish appropriate strategy to screen hepatotoxic markers in DBT extracts. In this study, three chemometric methods including PLSR, BP-ANN and cluster analysis were applied to discover the hepatotoxic markers from DBT extracts. Compared with the traditional toxicity-guided isolation process, the fingerprint-hepatotoxicity modeling approach showed its distinct advantages in efficiency, cost and general compatibility with holistic mode of HMs. The two principal discriminatory compounds, EEA and DIOB, were discovered as the hepatotoxic markers of DBT. Consequently, the combination of these two compounds was assigned as the candidate HECMs accounting for the whole hepatotoxicity of original DBT extracts. As shown in Fig. 4(A), the sum of DIOB and EEA correlated well with hepatotoxicity. In samples **S01**, **02**, 0**4**, **07**, **08**, **10**, **11**, **16** and **21**, low amount of DIOB and EEA associated with low serum ALT/AST levels. While in samples **S06, 09**, **13**, **14**, **15**, **17**, **18** and **20**, a similar trend was observed, *viz*. relatively higher contents of DIOB and EEA corresponded to more potent toxicity (Fig. 4(B)). The content-toxicity correlation implied that the combination of DIOB and EEA might be the candidate HECMs for the hepatotoxicity evaluation of DBT extract. Additionally, as listed in Table 2, the relatively higher VIP value and MIV of EEA indicated it was more potent than DIOB (1.798 *vs* 1.025, and 0.342 *vs* 0.255, respectively), which was consistent with a previous report^25^. Thus, compared with DIOB, EEA might play a major role in the DBT-induced liver injury.

Seen from Fig. 7(A), it was found that the mixtures of DIOB and EEA at identical amounts with those in S22–S24 showed nearly equivalent hepatotoxicities with corresponding DBT extracts, since all the 90% CI fell within the range of 70–143%^49^, demonstrating that candidate HECMs hepatotoxically equaled to original DBT extracts. Therefore, the chemical combination of EEA and DIOB was confirmed as the real HECMs for DBT.

In conclusion, a strategy based on the fingerprint-toxicity relationship modeling and hepatotoxic equivalence assessment was initially proposed for discovery and verification of HECMs from DBT. The chemical constituents in DBT extract was characterized by UHPLC-QTOF MS and a total of 40 compounds were identified or tentatively assigned. Based on the fingerprint-toxicity relationship modeling, EEA and DIOB were discovered as the main hepatotoxic markers. Furthermore, the chemical combination of those two markers was confirmed as HECMs which could account for the whole hepatotoxicity of original DBT extracts with 90% CI. Using DBT as a case study, this work provides not only a promising strategy for efficient discovery of potential hepatotoxic constituents from HMs, but also a rational terminology—HECMs for hepatotoxicity evaluation/prediction of HMs.

## Materials and Methods

### Chemicals and materials

A total of 24 batches of DBT samples (**S01**–**S24**) were collected from different regions in China. The voucher specimens, identified by Professor Hui-Jun Li of China Pharmaceutical University, were deposited in the State Key Laboratory of Natural Medicines, China Pharmaceutical University, Nanjing, China. The HPLC-grade solvents, acetonitrile, methanol and formic acid were purchased from Merck (Darmstadt, Germany), deionized water (18 MΩ cm) was prepared by distilled water through a Milli-Q system (Millipore, Milford, MA, USA). Other reagents and chemicals are of analytical grade. The reference standards of sucrose (**1**), shikimic acid (**2**), succinic acid (**4**), gallic acid (**6**), epigallocatechin (**7**), protocatechuic acid (**9**), catechin (**15**), epicatechin (**18**), caryatin (**26**), myricetin (**29**), quercetin (**36**) and kaempferol (**40**) were purchased from the National Institute for the Control of Pharmaceutical and Biological Products (Beijing, China) (purity >98%). Diosbulbin D (DIOD, **37**) was purchased from ChemFaces (Chengdu, China) (purity >98%). Diosbulbin M (DIOM, **28**), diosbulbin L (DIOL, **30**), diosbulbin C (DIOC, **32**), 3,5,3'-trimethoxyquercein (**33**), diosbulbin G (DIOG, **35**), DIOB, **38** and EEA, **39** were isolated from DBT in our laboratory. Their structures were identified by ESI-HRMS, ^1^H NMR and ^13^C NMR in comparison with the literature data, and purities were determined to be >98% by UHPLC-DAD analysis based on a peak area normalization method.

### Preparation of DBT extracts

The dried powder of DBT was soaked by refluxing with 80% ethanol (1:10, w/v) for 120 min and repeated three times. The ethanol extracts were combined to be filtered, concentrated under vacuum and then suspended in distilled water (1:1, v/v). The suspension was further extracted with the same volume of ethyl acetate for three times. The ethyl acetate layer was obtained and evaporated under vacuum, leading to the DBT extract. An aliquot of 10 mg DBT extract was dissolved in 1 mL of methanol and filtered through a 0.22 µm filter. The filtrate was injected for analysis.

### Chromatographic and mass spectrometric conditions

Chromatographic analysis was performed on an Agilent series 1290 UHPLC system equipped with a quaternary pump, a degasser, a diode array detector and a thermostated column compartment (Agilent Technologies, Palo Alto, CA, USA). Chromatographic separation was carried out at 25 °C on a Shim-pack VP-ODS column (4.6 mm × 250 mm, 5 μm). The mobile phase was a mixture of 0.1% formic acid in water (A) and acetonitrile (B) with a gradient elution as follows: 0 min, 96% (A); 20 min, 83% (A); 55 min, 46% (A); 65 min, 0% (A); 70 min, 0% (A). The flow rate was 0.8 mL/min, and the column temperature was set at 25 °C. The detection wavelength was 210 nm. Mass spectrometric analysis was performed on a 6530 QTOF mass spectrometer (Agilent Technologies, Santa Clara, CA, USA) equipped with electrospray ionization source in negative mode. The mass spectrometric conditions were as follows: nebulizer pressure, 35 psi; capillary voltage, 3500 V; fragmentor voltage, 135 V; drying gas flow, 10 L/min; drying gas temperature, 350 °C; sheath gas flow, 11 L/min; sheath gas temperature, 350 °C. The mass rang was recorded from *m/z* 100 to 1500 Da. Data acquisition was performed with MassHunter Workstation (Agilent Technologies, USA). The TOF mass spectrometer was calibrated every day before sample analysis using reference masses at *m*/*z* 121.0508 and 922.0098.

### Animal experiments

Male ICR mice (18–20 g) were purchased from Sino-British SIPPR/BK Lab Animal Ltd. (Shanghai, China). The mice were fed a standard laboratory diet and given free access to tap water, kept in a controlled room temperature (22 ± 1 °C), humidity (65 ± 5%), and a 12:12-h light/dark cycle for at least one week before treatment. Animal studies were conducted in accordance with the Provision and General Recommendation of Chinese Experimental Animals Administration Legislation and were approved by Department of Science and Technology of Jiangsu Province (license number: SYXK (SU) 2016-0011).

### Hepatotoxicity of DBT extracts

Mice were orally administered DBT extracts (2 g/kg, suspended in 0.5% CMC-Na, *n* = 10) for 36 h, and 0.5% sodium carboxymethyl cellulose (CMC-Na) was used as a vehicle control (*n* = 10)^25,50^. They were fasted from food, but no water 12 h prior to the administration of the test suspension. Blood was collected from the eyeball for measurement of ALT, AST. Serum ALT and AST activities were measured on Cobas 8000 modular analyzer (Basel, Switzerland). Liver tissues were fixed in 10% neutral buffered formalin, paraffin processed, and sectioned at 3 μm. For histological evaluation, the tissue sections were stained with hematoxylin and eosin (H&E).

### Establishing of UHPLC fingerprint

All DBT samples were chemically profiled under the above mentioned chromatographic and mass spectrometric conditions. The fingerprints of 21 batches of samples (**S01**–**S21**) were matched automatically using Similarity Evaluation System for Chromatographic Fingerprint of Traditional Chinese Medicine (2012 Version, Committee of Chinese Pharmacopeia). The simulative mean chromatogram as a representative standard for those fingerprints was calculated and generated automatically by median method. Based on careful comparison of UV, MS/MS spectra and relative retention time, peaks detected in all fingerprints were defined as “common characteristic peaks” and structurally elucidated. Similarity values between each two chromatographic fingerprints were then determined using the above mentioned officially recommended software.

### Quantitative analysis of the major diterpenoid lactones

The contents of DIOB and EEA were quantified by means of the external standard method. Prior to quantification, the developed UHPLC method was fully validated in terms of specificity, linearity, limit of detection (LOD), limit of quantitation (LOQ), precision (*i*.*e*. repeatability, intra-day and inter-day variability), stability, repeatability and accuracy.

### Statistical analysis

Results were expressed as the mean ± standard deviation (SD) for continuous variables and as the number (percent) for categorical variables. To maximize identification the fingerprint-toxicity relationship between groups, PLSR model was applied using SIMCA version 14.0.1 (Umetrics AB, Umea, Sweden). In addition to the multivariate statistical method, the BP-ANN model was also employed to correlate fingerprints with hepatotoxicity using Matlab R2016a (Mathworks, Natick, USA). Heat maps and hierarchical cluster analyses were conducted using MeV version 4.6.0. Statistical analyses were performed using SPSS software version 19.0 (IBM Corp., Armonk, USA). An adjusted *p* value < 0.05 was considered statistically significant.

### Hepatotoxicity evaluation between candidate HECMs and original DBT extracts

Hepatotoxic equivalence was evaluated by calculating 90% CI of the ratio between the toxicities of candidate HECMs and original DBT extracts (two one-sided *t* test), the equivalent relationship of the 90% CI was calculated by the following equation:

2 $$[\exp (\overline{{Y}_{B}}-\overline{{Y}_{H}}-{t}_{0.95,{n}_{1}+{n}_{2}-2}{\hat{\sigma }}_{W}\sqrt{\frac{1}{2}(\frac{1}{{n}_{1}}+\frac{1}{{n}_{2}})}),\exp (\overline{{Y}_{B}}-\overline{{Y}_{H}}+{t}_{0.95,{n}_{1}+{n}_{2}-2}{\hat{\sigma }}_{W}\sqrt{\frac{1}{2}(\frac{1}{{n}_{1}}+\frac{1}{{n}_{2}})})]\subset (0.7,1.43)$$

Where $\overline{{Y}_{B}}$and $\overline{{Y}_{H}}$ are the least squares means of the candidate HECMs and original DBT extracts treatment, ${\hat{\sigma }}_{W}^{2}$ is the mean square, from Analysis of Variance (ANOVA) after logarithmic transformation, and ${t}_{0.95,{n}_{1}+{n}_{2}-2}$is the 0.95 quantile of the central *t*-distribution with *n*_1_ + *n*_2_-2 degrees of freedom^26^. If the 90% CI of relative hepatotoxicity compared to original DBT fell within the range of 70–143%^49^, the candidate HECMs were considered to be hepatotoxic equivalent with original DBT extracts. Three batches of additional DBT samples from Sichuan (**S22**), Anhui (**S23**) and Hubei (**S24**) were chosen to evaluate the hepatotoxic equivalence between candidate HECMs and original DBT extracts. The mixtures of DIOB and EEA were dissolved in 0.5% CMC-Na solution at a dose equivalent to that of the two tested compounds found in the DBT extracts. The mice were sacrificed 36 h after the administration, and the serum ALT and AST activities were used to assess the hepatotoxicity. Meanwhile, the histological samples were also examined.

## Electronic supplementary material

Supplementary information
