# Supplementary material for: Discovery of Hepatotoxic Equivalent Combinatorial Markers from Dioscorea bulbifera tuber by Fingerprint-Toxicity Relationship Modeling
Source: Sci Rep. 2018 Jan 11;8:462. doi: 10.1038/s41598-017-18929-z (PMC5764974; doi:10.1038/s41598-017-18929-z)
Supplement: Supplementary file 1 — Supplementary information [file 41598_2017_18929_MOESM1_ESM.doc]

**Supplementary information**

**Discovery of Hepatotoxic Equivalent Combinatorial Markers from *Dioscorea bulbifera* tuber by Fingerprint-Toxicity Relationship Modeling**

Wei Shi, Cai Zhang, Dongsheng Zhao, Lingli Wang, Ping Li* & Huijun Li*

State Key Laboratory of Natural Medicines, China Pharmaceutical University,

Nanjing, China

Corresponding authors:

Ping Li, Ph D E-mail: liping2004@126.com.

Huijun Li, PhD E-mail: cpuli@163.com.

State Key Laboratory of Natural Medicines, China Pharmaceutical University, No. 24

Tongjia Lane, Nanjing 210009, China.

Tel.: +86 25 83271382; Fax: +86 25 83271379.

**Figure S1.** The total ion chromatogram of DBT extract by UHPLC-QTOF MS analysis in positive ion mode (**S01**). *: HEMCs

**
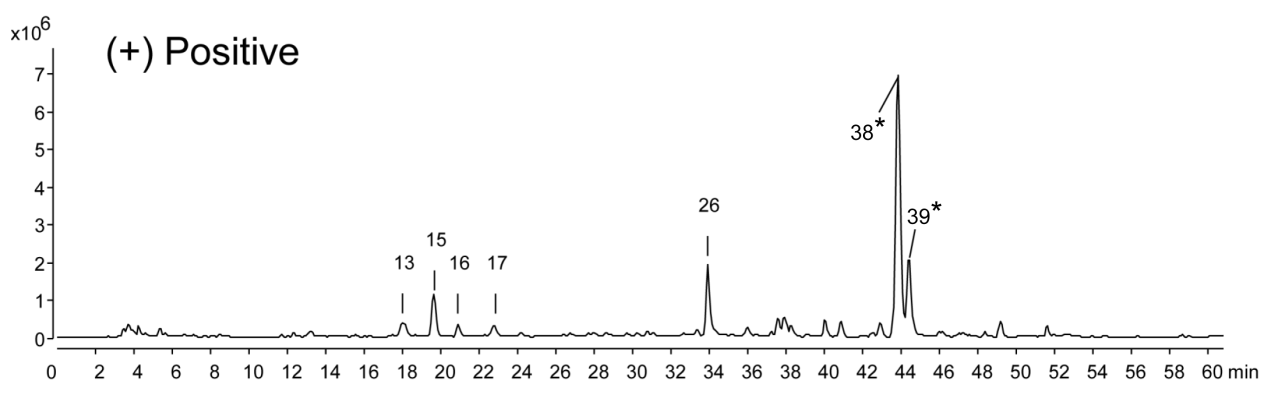
**

**Table S1**. Similarly values of 21 batches of DBT extracts (**S01** – **S21**).

| **Sample** | **S01** | **S02** | **S03** | **S04** | **S05** | **S06** | **S07** | **S08** | **S09** | **S10** | **S11** | **S12** | **S13** | **S14** | **S15** | **S16** | **S17** | **S18** | **S19** | **S20** | **S21** | **Reference** |
| --- | --- | --- | --- | --- | --- | --- | --- | --- | --- | --- | --- | --- | --- | --- | --- | --- | --- | --- | --- | --- | --- | --- |
| **S01** | 1.000 | 0.940 | 0.941 | 0.99 | 0.974 | 0.993 | 0.972 | 0.982 | 0.975 | 0.939 | 0.953 | 0.99 | 0.976 | 0.981 | 0.965 | 0.926 | 0.987 | 0.942 | 0.986 | 0.980 | 0.963 | 0.981 |
| **S02** | 0.940 | 1.000 | 0.992 | 0.956 | 0.934 | 0.964 | 0.982 | 0.972 | 0.973 | 0.984 | 0.992 | 0.953 | 0.982 | 0.961 | 0.950 | 0.992 | 0.973 | 0.872 | 0.960 | 0.968 | 0.992 | 0.987 |
| **S03** | 0.941 | 0.992 | 1.000 | 0.959 | 0.943 | 0.963 | 0.970 | 0.969 | 0.978 | 0.996 | 0.979 | 0.960 | 0.990 | 0.962 | 0.947 | 0.981 | 0.975 | 0.858 | 0.967 | 0.982 | 0.989 | 0.987 |
| **S04** | 0.990 | 0.956 | 0.959 | 1.000 | 0.971 | 0.995 | 0.979 | 0.987 | 0.985 | 0.960 | 0.961 | 0.980 | 0.984 | 0.971 | 0.957 | 0.944 | 0.983 | 0.905 | 0.974 | 0.986 | 0.98 | 0.986 |
| **S05** | 0.974 | 0.934 | 0.943 | 0.971 | 1.000 | 0.979 | 0.943 | 0.986 | 0.962 | 0.930 | 0.926 | 0.974 | 0.971 | 0.983 | 0.991 | 0.907 | 0.983 | 0.953 | 0.978 | 0.970 | 0.956 | 0.972 |
| **S06** | 0.993 | 0.964 | 0.963 | 0.995 | 0.979 | 1.000 | 0.986 | 0.993 | 0.983 | 0.958 | 0.969 | 0.989 | 0.987 | 0.986 | 0.974 | 0.951 | 0.995 | 0.930 | 0.986 | 0.987 | 0.983 | 0.993 |
| **S07** | 0.972 | 0.982 | 0.970 | 0.979 | 0.943 | 0.986 | 1.000 | 0.981 | 0.976 | 0.966 | 0.992 | 0.967 | 0.978 | 0.965 | 0.950 | 0.984 | 0.982 | 0.902 | 0.967 | 0.972 | 0.991 | 0.990 |
| **S08** | 0.982 | 0.972 | 0.969 | 0.987 | 0.986 | 0.993 | 0.981 | 1.000 | 0.983 | 0.960 | 0.972 | 0.976 | 0.986 | 0.982 | 0.985 | 0.958 | 0.991 | 0.941 | 0.980 | 0.982 | 0.988 | 0.993 |
| **S09** | 0.975 | 0.973 | 0.978 | 0.985 | 0.962 | 0.983 | 0.976 | 0.983 | 1.000 | 0.979 | 0.974 | 0.971 | 0.990 | 0.967 | 0.955 | 0.962 | 0.981 | 0.899 | 0.977 | 0.990 | 0.986 | 0.990 |
| **S10** | 0.939 | 0.984 | 0.996 | 0.960 | 0.930 | 0.958 | 0.966 | 0.960 | 0.979 | 1.000 | 0.975 | 0.954 | 0.987 | 0.949 | 0.928 | 0.978 | 0.966 | 0.837 | 0.959 | 0.982 | 0.985 | 0.981 |
| **S11** | 0.953 | 0.992 | 0.979 | 0.961 | 0.926 | 0.969 | 0.992 | 0.972 | 0.974 | 0.975 | 1.000 | 0.954 | 0.976 | 0.956 | 0.941 | 0.993 | 0.972 | 0.887 | 0.961 | 0.967 | 0.990 | 0.986 |
| **S12** | 0.990 | 0.953 | 0.960 | 0.98 | 0.974 | 0.989 | 0.967 | 0.976 | 0.971 | 0.954 | 0.954 | 1.000 | 0.986 | 0.993 | 0.972 | 0.931 | 0.994 | 0.926 | 0.996 | 0.987 | 0.965 | 0.984 |
| **S13** | 0.976 | 0.982 | 0.990 | 0.984 | 0.971 | 0.987 | 0.978 | 0.986 | 0.990 | 0.987 | 0.976 | 0.986 | 1.000 | 0.984 | 0.969 | 0.965 | 0.992 | 0.900 | 0.989 | 0.997 | 0.989 | 0.996 |
| **S14** | 0.981 | 0.961 | 0.962 | 0.971 | 0.983 | 0.986 | 0.965 | 0.982 | 0.967 | 0.949 | 0.956 | 0.993 | 0.984 | 1.000 | 0.990 | 0.935 | 0.996 | 0.946 | 0.995 | 0.979 | 0.967 | 0.986 |
| **S15** | 0.965 | 0.95 | 0.947 | 0.957 | 0.991 | 0.974 | 0.950 | 0.985 | 0.955 | 0.928 | 0.941 | 0.972 | 0.969 | 0.990 | 1.000 | 0.923 | 0.985 | 0.965 | 0.980 | 0.960 | 0.959 | 0.975 |
| **S16** | 0.926 | 0.992 | 0.981 | 0.944 | 0.907 | 0.951 | 0.984 | 0.958 | 0.962 | 0.978 | 0.993 | 0.931 | 0.965 | 0.935 | 0.923 | 1.000 | 0.957 | 0.852 | 0.938 | 0.952 | 0.988 | 0.976 |
| **S17** | 0.987 | 0.973 | 0.975 | 0.983 | 0.983 | 0.995 | 0.982 | 0.991 | 0.981 | 0.966 | 0.972 | 0.994 | 0.992 | 0.996 | 0.985 | 0.957 | 1.000 | 0.939 | 0.995 | 0.989 | 0.984 | 0.996 |
| **S18** | 0.942 | 0.872 | 0.858 | 0.905 | 0.953 | 0.930 | 0.902 | 0.941 | 0.899 | 0.837 | 0.887 | 0.926 | 0.900 | 0.946 | 0.965 | 0.852 | 0.939 | 1.000 | 0.94 | 0.899 | 0.890 | 0.919 |
| **S19** | 0.986 | 0.960 | 0.967 | 0.974 | 0.978 | 0.986 | 0.967 | 0.980 | 0.977 | 0.959 | 0.961 | 0.996 | 0.989 | 0.995 | 0.980 | 0.938 | 0.995 | 0.940 | 1.000 | 0.988 | 0.968 | 0.988 |
| **S20** | 0.980 | 0.968 | 0.982 | 0.986 | 0.970 | 0.987 | 0.972 | 0.982 | 0.990 | 0.982 | 0.967 | 0.987 | 0.997 | 0.979 | 0.960 | 0.952 | 0.989 | 0.899 | 0.988 | 1.000 | 0.981 | 0.991 |
| **S21** | 0.963 | 0.992 | 0.989 | 0.980 | 0.956 | 0.983 | 0.991 | 0.988 | 0.986 | 0.985 | 0.990 | 0.965 | 0.989 | 0.967 | 0.959 | 0.988 | 0.984 | 0.890 | 0.968 | 0.981 | 1.000 | 0.995 |
| **Reference** | 0.981 | 0.987 | 0.987 | 0.986 | 0.972 | 0.993 | 0.990 | 0.993 | 0.990 | 0.981 | 0.986 | 0.984 | 0.996 | 0.986 | 0.975 | 0.976 | 0.996 | 0.919 | 0.988 | 0.991 | 0.995 | 1.000 |

**Table S2. Common peak areas in fingerprints of 21 batches of DBT extracts (S01 – S21).**

| **Sample** | **Location** | **Peak 1** | **Peak 2** | **Peak 3** | **Peak 4** | **Peak 5** | **Peak 6** | **Peak 7** | **Peak 8** | **Peak 9(*)** | **Peak 10(*)** |
| --- | --- | --- | --- | --- | --- | --- | --- | --- | --- | --- | --- |
| **S01** | Yunnan | 2132 | 4717 | 15413 | 31114 | 12410 | 6066 | 7576 | 11606 | 4838 | 1499 |
| **S02** | Gansu | 8311 | 21029 | 47440 | 46833 | 28410 | 21234 | 16320 | 17943 | 5644 | 4442 |
| **S03** | Sichuan | 6621 | 17510 | 36900 | 40876 | 18297 | 15904 | 11341 | 11143 | 647 | 6539 |
| **S04** | Guizhou | 4106 | 7009 | 25101 | 41426 | 19113 | 7370 | 6672 | 11189 | 5147 | 1149 |
| **S05** | Jiangxi | 2265 | 3307 | 17318 | 35793 | 13543 | 15481 | 6627 | 8332 | 3733 | 2976 |
| **S06** | Hunan | 6711 | 8172 | 28538 | 48166 | 23148 | 12510 | 10172 | 16852 | 5812 | 3380 |
| **S07** | Fujian | 9811 | 15258 | 35624 | 45961 | 27193 | 12558 | 10989 | 22980 | 5875 | 460 |
| **S08** | Yunnan | 3902 | 6816 | 24863 | 37654 | 17376 | 15002 | 7274 | 12377 | 5006 | 136 |
| **S09** | Guangdong | 7474 | 11463 | 30948 | 42977 | 14387 | 10805 | 10742 | 10764 | 1169 | 8166 |
| **S10** | Anhui | 4983 | 17706 | 33397 | 37625 | 15212 | 10645 | 8731 | 8968 | 1424 | 5570 |
| **S11** | Zhejiang | 8009 | 20907 | 46552 | 47871 | 27176 | 16526 | 15593 | 25630 | 8522 | 1157 |
| **S12** | Guangxi | 2442 | 6242 | 16782 | 31829 | 13419 | 7788 | 10469 | 10886 | 2307 | 5801 |
| **S13** | Sichuan | 3740 | 9747 | 26602 | 36375 | 14833 | 10975 | 9759 | 10210 | 2527 | 5318 |
| **S14** | Jiangsu | 3038 | 6961 | 22582 | 39269 | 18508 | 15572 | 12674 | 14261 | 4831 | 8484 |
| **S15** | Hunan | 2784 | 4973 | 21161 | 37733 | 17723 | 21107 | 10711 | 12439 | 6217 | 4934 |
| **S16** | Hebei | 14113 | 32720 | 56520 | 54806 | 34811 | 22654 | 14878 | 26371 | 6125 | 110 |
| **S17** | Sichuan | 4718 | 9281 | 23881 | 39597 | 17942 | 13999 | 10756 | 14511 | 3976 | 5407 |
| **S18** | Fujian | 471 | 777 | 5356 | 14674 | 5087 | 8479 | 3849 | 8235 | 4452 | 372 |
| **S19** | Guangxi | 2210 | 6075 | 16304 | 28494 | 10104 | 9238 | 9937 | 10309 | 2886 | 4639 |
| **S20** | Guangdong | 4267 | 8621 | 25348 | 37467 | 12249 | 8882 | 8561 | 10672 | 2207 | 5337 |
| **S21** | Gansu | 8019 | 15340 | 36469 | 44134 | 23874 | 15751 | 9309 | 14660 | 4432 | 959 |

*: HEMCs.
